# Sphingosine-1 phosphate receptor 1 contributes to central sensitization in recurrent nitroglycerin-induced chronic migraine model

**DOI:** 10.1186/s10194-022-01397-w

**Published:** 2022-02-10

**Authors:** Qi Pan, Yunfeng Wang, Ruimin Tian, Qianwen Wen, Guangcheng Qin, Dunke Zhang, Lixue Chen, Yixin Zhang, Jiying Zhou

**Affiliations:** 1 Department of Neurology, The First Affiliated Hospital of Chongqing Medical University, 1st You Yi Road, Yuzhong District, Chongqing, 400016 China; 2 Department of Neurology, Nanchong Central Hospital, The Second Clinical Medical College, North Sichuan Medical College, Nanchong, China; 3 Laboratory Research Center, The First Affiliated Hospital of Chongqing Medical University, Chongqing, China

**Keywords:** Chronic migraine, Central sensitization, S1PR1, Microglia, STAT3

## Abstract

**Background:**

Central sensitization is an important pathophysiological mechanism of chronic migraine (CM), and microglia activation in trigeminocervical complex (TCC) contributes to the development of central sensitization. Emerging evidence implicates that blocking sphingosine-1-phosphate receptor 1 (S1PR1) can relieve the development of chronic pain and inhibit the activation of microglia. However, it is unclear whether S1PR1 is involved in the central sensitization of CM. Therefore, the purpose of this study is to explore the role of S1PR1 and its downstream signal transducers and activators of transcription 3 (STAT3) signaling pathway in the CM, mainly in inflammation.

**Methods:**

Chronic intermittent intraperitoneal injection of nitroglycerin (NTG) established a mouse model of CM. First, we observed the changes and subcellular localization of S1PR1 in the trigeminocervical complex (TCC). Then, W146, a S1PR1 antagonist; SEW2871, a S1PR1 agonist; AG490, a STAT3 inhibitor were applied by intraperitoneal injection to investigate the related molecular mechanism. The changes in the number of microglia and the expression of calcitonin gene-related peptide (CGRP) and c-fos in the TCC site were explored by immunofluorescence. In addition, we studied the effect of S1PR1 inhibitors on STAT3 in lipopolysaccharide-treated BV-2 microglia.

**Results:**

Our results showed that the expression of S1PR1 was increased after NTG injection and S1PR1 was colocalized with in neurons and glial cells in the TCC. The S1PR1 antagonist W146 alleviated NTG-induced hyperalgesia and suppressed the upregulation of CGRP, c-fos and pSTAT3 in the TCC. Importantly, blocking S1PR1 reduced activation of microglia. In addition, we found that inhibiting STAT3 signal also attenuated NTG-induced basal mechanical and thermal hyperalgesia.

**Conclusions:**

Our results indicate that inhibiting S1PR1 signal could alleviate central sensitization and inhibit microglia activity caused by chronic NTG administration via STAT3 signal pathway, which provide a new clue for the clinical treatment of CM.

**Supplementary Information:**

The online version contains supplementary material available at 10.1186/s10194-022-01397-w.

## Background

Migraine is a chronic neurological disease that currently affects more than one billion people worldwide [1]. Approximately 2.5% of patients with episodic migraine will progress to chronic migraine (CM) per year, which further increases the economic burden of individuals and society [2]. CM is defined as headaches ≥ 15 days per month and monthly migraine attacks ≥ 8 days for at least 3 months [3]. Limited treatment options and poor treatment response make the treatment of CM still challenging [4]. The exploration of the pathophysiological mechanism might suggest new therapeutic targets for CM.

Central sensitization is an important pathophysiological mechanism of CM, manifesting as a prolonged excitability of the second-order neurons in medullary horn (trigeminocervical complex, TCC) or third-order thalamic neurons [5, 6]. Clinically, patients with CM often manifest cephalic and extracephalic allodynia [7]. Emerging evidence suggests that central sensitization could be driven by activation of glial cells and release of pro-inflammatory cytokines and chemokines [8, 9]. Our previous studies found that activation of microglia and up-regulation of microglial purinoceptors (P2X4R, P2X7R and P2Y12R) in TCC area contribute to the development of central sensitization in the CM [10–12].

Sphingosine-1-phophate (S1P), a metabolite of sphingomyelin, is a biologically active lipid with important physiological functions, widely expressed in blood, red blood cells and central nervous system [13]. As an extracellular ligand, S1P binds to S1P receptors (S1PR1-5) and activates many intracellular signaling pathways to participate in the regulation of energy homeostasis, spinal nociceptive processing and inflammation [14, 15]. A growing body of evidence implicates activation of S1PR1 in the spinal cord contributes to the development of mechano-allodynia in a variety of neuropathic pain models, including paclitaxel-induced neuropathic pain, opioid-induced hyperalgesia [16, 17]. In vitro studies have found that S1PR1 is expressed in primary microglia, and inhibition of S1PR1 signal can block the activation of microglia and subsequent release of inflammatory mediators [18, 19]. In addition, some studies have shown that blocking S1PR1 signaling promotes the neuroprotective effect of microglia in a variety of central nervous system diseases [20, 21]. However, it is unclear whether S1PR1 is involved in the central sensitization of CM. Thus, our study aims to explore the role of S1PR1 in chronic nitroglycerin (NTG) model, with focus on inflammation.

S1PR1 is a kind of G-protein-coupled receptor that can induce the production of signal transducers and activators of transcription 3 (STAT3) by activating tyrosine kinases and serine/threonine kinases [22]. STAT3 is an important transcription factor that participates in cell growth, proliferation, differentiation and immune regulation [23]. In multiple pain models, STAT3 signaling pathway was involved in the regulation of microglia activation and neuroinflammation [24, 25]. Based on the above findings, we hypothesize that inhibiting the activity of S1PR1 in TCC area could alleviate the central sensitization and microglia activation caused by chronic nitroglycerin administration via STAT3 pathway.

## Materials and methods

### Animals

All experiments were carried out in accordance with the Guide for the Care and Use of Laboratory Animals and approved by the Animal Care and Use Committee at Chongqing Medical University in China. Adult male C57BL/6 J mice (20-25 g) were used in this study. As described in our previous studies, mice were randomly assigned to different experimental groups and the detailed randomization process was as follows: (1) First, mice were numbered, and the numbers were entered in the Excel table in ascending order. (2) Next, using the ‘rand ()’ function of the Excel software to create a new set of random numbers, and rearrange them in ascending order. (3) According to the new ranking, mice were assigned to different experimental groups [10, 26]. All mice were housed under standard conditions with 12 h light/dark cycle, controlled room temperature, enough water and food. Before all experiments started, mice were given one week to adapt to the experimental environment. All efforts were made to reduce the suffering of mice and minimize the number of animals. The sample size of this study was determined based on previous studies [27, 28].

### Chronic migraine model and drug administration

A mouse model of CM established by repeated intraperitoneal (*i.p.*) injections of NTG has been described in previous studies [27, 29]. In brief, a stock solution of 5 mg/ml NTG (Runhong Pharm, China) containing 30% propylene glycol, 30% alcohol and water was freshly diluted in 0.9% saline at 1 mg/ml before each injection. The vehicle control was 0.9% saline. Previous studies found that there was no significant difference in mechanical thresholds between 0.9% saline and the solution in which NTG was dissolved [27, 29]. Mice received 10 mg/kg NTG or saline (*i.p.*) every other day over 9 days.

W146 (3-amino-4-(3-hexylphenylamino)-4-oxobutyl phosphonic acid), a selective S1PR1 antagonist, was purchased from Cayman Chemical (Ann Arbor, MI). W146 was dissolved in 2% dimethyl sulfoxide (DMSO) in 0.9% saline, and an equal volume of solvent was used as the vehicle (VEH). W146 (1 mg/kg) or vehicle was administered (*i.p.*, 100 µl) 15 min before NTG inject for 9 days [30]. It has been reported that W146 is used for central nervous system diseases by intraperitoneal injection [30, 31]**.** A specific agonist, SEW2871(5-(4-phenyl-5-(trifluoromethyl)-2-thienyl)-3-(3-(trifluoromethyl) phenyl)-1,2,4-oxadiazole), was purchased from Cayman Chemical (MI, USA). SEW2871(20 mg/kg) was administrated (*i.p*., 200ul) prior to NTG injections for 9 days [16]. The vehicle was 5% DMSO/0.5methylcellulose/saline. To further elucidate the effects of STAT3, AG490 (Selleck, Texas, USA) was chosen based on previous studies. AG490 was first dissolved in 5% DMSO to prepare stock solution and stored at -20 °C. On the day of the experiment, 40% PEG300, 5%Tween 80 and 50% double distilled water were added. An equal volume of solvent was used as the vehicle. AG490 (20 mg/kg, *i.p*, 200 ul) were injected prior to the NTG for 9 days. The body weight of each mouse was recorded before administration, and no obvious side effects were found.

### Behavioral tests

Cutaneous allodynia, a biomarker of trigeminothalamic pathways sensitization, is evaluated in CM animal models by measuring mechanical and thermal sensitivity tests [32, 33]. All behavioral tests were performed under low-light conditions, two researchers remained blind to the experimental mice. Before each behavioral test, mice were acclimatized in the testing apparatus about 30 min. Baseline behavioral tests were performed before daily administration of drug or saline, and post-treatment behavioral responses were completed two hours after the NTG injection.

As previously reported, von Frey filaments were used to measure the periorbital and hindpaw mechanical sensitivity through the ‘up and down’ method [10, 27]. For periorbital mechanical threshold, the mouse was put into a 4 oz. cup and adapted 15 min. The bending force of filament was from 0.04 to 1* g*. For hindpaw mechanical threshold, 0.4 g of von Frey hair was firstly used to stimulate the hind paw foot surface 5 times with an interval of 30 s. If there were three or more positive reactions in 5 times, such as a lifting, licking or shaking of the paw, the stimulation intensity was reduced (the minimum was 0.04 g). Otherwise, the stimulation intensity was increased (up to 2 g).

Thermal nociceptive thresholds were measured using a plantar test apparatus (Techman PL-200, Chengdu, China). The heat intensity was determined based on the intensity that allowed an average withdrawal latency of 10–15 s in normal animals [10, 11]. The cut-off time was set at 20 s. The withdrawal latencies were recorded automatically when mice lifted their paws in a quiet state. The same hind paw of each mouse was measured repeatedly three times with an interval of 5 min, and the average was defined.

### Quantitative real-time polymerase chain reaction (qRT-PCR)

Detailed process of qRT-PCR has been elaborated in previous studies [26, 34]. After the mouse was deeply anesthetized with 1% sodium pentobarbital, the TCC tissue was quickly removed and placed in liquid nitrogen for temporary storage. Total RNA was extracted using an RNAiso Plus reagent (TaKaRa, Dalian), and the OD_260_ was measured by NanoDrop spectrophotometer (Thermo, USA). For reverse transcription, the cDNA was synthesized using the PrimeScript™ RT reagent kit (Takara, Tokyo, Japan). Quantitative polymerase chain reaction was performed on a CFX96 Touch thermocycler (Bio-Rad) using the SYBRVR Premix Ex TaqTM II (Takara) according to the instructions. The sequences of forward and reverse primers were described as follows:

*S1PR1*: 5’-CTGACCTTCCGCAAGAACATCTCC-3’(forward), 5’- CCCAGCAGGCAATGAAGACACTC-3’(reverse);

*GAPDH*: 5’-ATGACTCTACCC ACGGCA AGC T-3’ (forward), 5’-GGATGCAGGGATGATGTTCT-3’(reverse). A relative change in gene expression was calculated by the 2^–ΔΔCT^ method. GAPDH (glyceraldehyde-3-phosphate dehydrogenase) was used as a control.

### Western blot analysis

At the end of behavioral testing, TCC segments (from obex to C2 cervical spinal cord) were quickly removed and stored at -80 °C. After the tissues were weighed, radioimmunoprecipitation assay buffer (Beyotime, Shanghai, China), phenylmethylsulfonyl fluoride (Beyotime, Shanghai, China) and phosphatase inhibitor (MedChemExpress, USA) were added and homogenized for 1 h at 4 °C. The protein concentration was determined by a BCA protein assay kit (Beyotime, Shanghai, China). Equal amounts of protein samples (40 μg) were separated by 10% sodium dodecyl sulfate polyacrylamide (SDS-PAGE) gels (Beyotime, Shanghai, China), and then transferred to 0.45 μm polyvinylidene fluoride (Millipore, USA) membrane at 100 V (Bio-Rad, California, USA). These membranes were blocked in 5% non-fat dry milk for 2 h at room temperature, followed by incubation with the following antibodies overnight at 4 °C: rabbit anti-pSTAT3, mouse anti-STAT3, rabbit anti-iNOS and mouse anti-GAPDH (See Table 1 for details). Next day, membranes were washed with TBST and incubated in a horseradish peroxidase-conjugated secondary antibody (goat anti-rabbit, 1:9000; goat anti-mouse, 1:5000; ZSGB-BIO, China) at room temperature for 1 h. Membranes were visualized (Fusion, Germany) using the ECL Plus kit (ZSGB-BIO, China).

**Table 1:** All antibodies used in western blotting and immunofluorescence staining

Antibody	Manufacturer	Host	Dilution
**For western blotting analysis**			
S1PR1	Abcam, UK	Rabbit	1:1000
pSTAT3	Cell Signaling Technology, USA	Rabbit	1: 2000
STAT3	Cell Signaling Technology, USA	Mouse	1: 1000
iNOS	Proteintech, China	Rabbit	1: 1000
GAPDH	ZEN-BIOSCIENCE, China	Mouse	1: 5000
Anti-mouse IgG (HRP)	ZEN-BIOSCIENCE, China	Goat	1: 9000
Anti-rabbit IgG (HRP)	ZEN-BIOSCIENCE, China	Goat	1: 5000
**For immunofluorescence staining**			
S1PR1	Abcam, UK	Rabbit	1: 400
Iba1	Wako, Japan	Rabbit	1: 400
Iba1	Wako, Japan	Goat	1: 1000
NeuN	Cell Signaling Technology, USA	Mouse	1: 400
GFAP	Santa Cruz, USA	Mouse	1: 200
CGRP	Santa Cruz, USA	Mouse	1: 100
c-fos	Novus Biologicals, USA	Rabbit	1: 5000
Alexa Fluor 488 goat anti-mouse IgG	Beyotime, China	Goat	1: 500
Cy3-conjugated goat anti-rabbit IgG	Beyotime, China	Goat	1: 500
DyLight 488 rabbit anti-goat IgG	Abbkine, USA	Rabbit	1: 500

### BV-2 microglial cells culture and treatment

BV2 microglial cells (obtained from institute of Basic Medicine and Cancer, Chinese Academy of Sciences) were cultured in Dulbecco’s modified Eagle’s medium supplemented with 10% foetal bovine serum, 1% penicillin–streptomycin solution at 37 °C in a humidified environment containing 5% CO2. BV2 cells were stimulated with lipopolysaccharides (LPS, 1 µg/ml, Sigma-Aldrich, MO, USA) to assess the expression of pSTAT3 at different time points, i.e., 0 h, 2 h, 6 h and 12 h. To explore the molecular mechanism of microglia S1PR1, BV2 cells were given W146 (250 nm) for 1 h before LPS stimulation. The dosage of these drugs was determined based on previous studies [11, 19, 35].

### Western blot analysis

BV2 cells were seeded in 100 mm culture dishes for western blot. Cultured BV2 cells were homogenized in radioimmunoprecipitation assay buffer buffer containing phenylmethylsulfonyl fluoride and phosphatase inhibitors. Samples were kept on ice for 30 min followed by centrifugation at 12000* g* at 4 °C for 15 min. Cell protein concentration was determined by BCA kit (Beyotime, Shanghai, China). The following experimental process was described above.

### Immunofluorescence staining

Frozen brain sections were mainly used for immunofluorescence as previously reported [10, 11, 28]. Mice were deeply anaesthetized with 10% chloral hydrate and transcardially perfused with 60 ml ice cold 0.1 M PBS, followed by 60 ml 4% paraformaldehyde (pH = 7.4). The whole brain and upper neck (C1-C2) were removed and postfixed for 24 h at 4 °C. Then, the brain tissue was dehydrated in 20% and 30% sucrose solutions sequentially. The hindbrain tissue was quickly frozen in 2-methylbutane (Aladdin, Shanghai, China) and then embedded. According to the Mouse Brain Reference Atlas, the segment of TCC was continuously cross-cut to a thickness of 15 μm (Leica, Germany) and stored in -80 °C. After heat-mediated antigen retrieval with sodium citrate (Beyotime, Shanghai, China), the sections were permeabilized with 0.3% Triton X-100 (Beyotime, Shanghai, China) for 10 min and then blocked with 5% goat serum for 30 min at 37 °C. The primary antibody was incubated overnight for 4 °C, including the following antibodies: Iba-1 antibody, NeuN antibody, GFAP antibody, CGRP antibody and c-fos antibody (see Table1 in detail). The next day, the sections were incubated with Cy3-conjugated or fluorescence-conjugated secondary antibodies (1:500; Beyotime, China) for 1 h at 37 °C. For double immunofluorescence, the sections were incubated with a mixture of primary or secondary antibodies from two different sources. 4′,6-diamidino-2-phenylindole (Beyotime, China) was used to counterstain the nuclei for 10 min at 37 °C. The stained sections were examined and captured with a confocal microscope (LSM800, ZEISS, Germany). To quantify c-fos, Iba1 positive cells profiles in the TCC, four to six sections from each mouse were randomly selected. An image centered on the superficial layer (laminas I–IV) of the TCC was acquired under a 100 × objective, and all positively stained cells were counted with Image J software (Wayne Rasband, National Institute of Health, USA). The mean optical density of CGRP was analyzed using Image J software with a 100 × objective.

### Statistical analysis

All data were statistically analyzed and graphed by GraphPad Prism version 7.0 (GraphPad Software Inc., San Diego, USA). All results were expressed as mean ± SEM. The normality of these results was examined by Kolmogorov–Smirnov test. The comparison between two samples were determined by Student’s *t* test. The differences among three or more groups were determined by one-way ANOVA followed by Tukey post hoc test. Two-way analysis of variance followed by Tukey / Bonferroni post hoc test was used in behavioral statistics. *P* < 0.05 was defined as statistically significant.

## Results

### Upregulation of S1PR1 expression at mRNA and protein levels in the TCC after chronic NTG injection

**Fig. 1:**
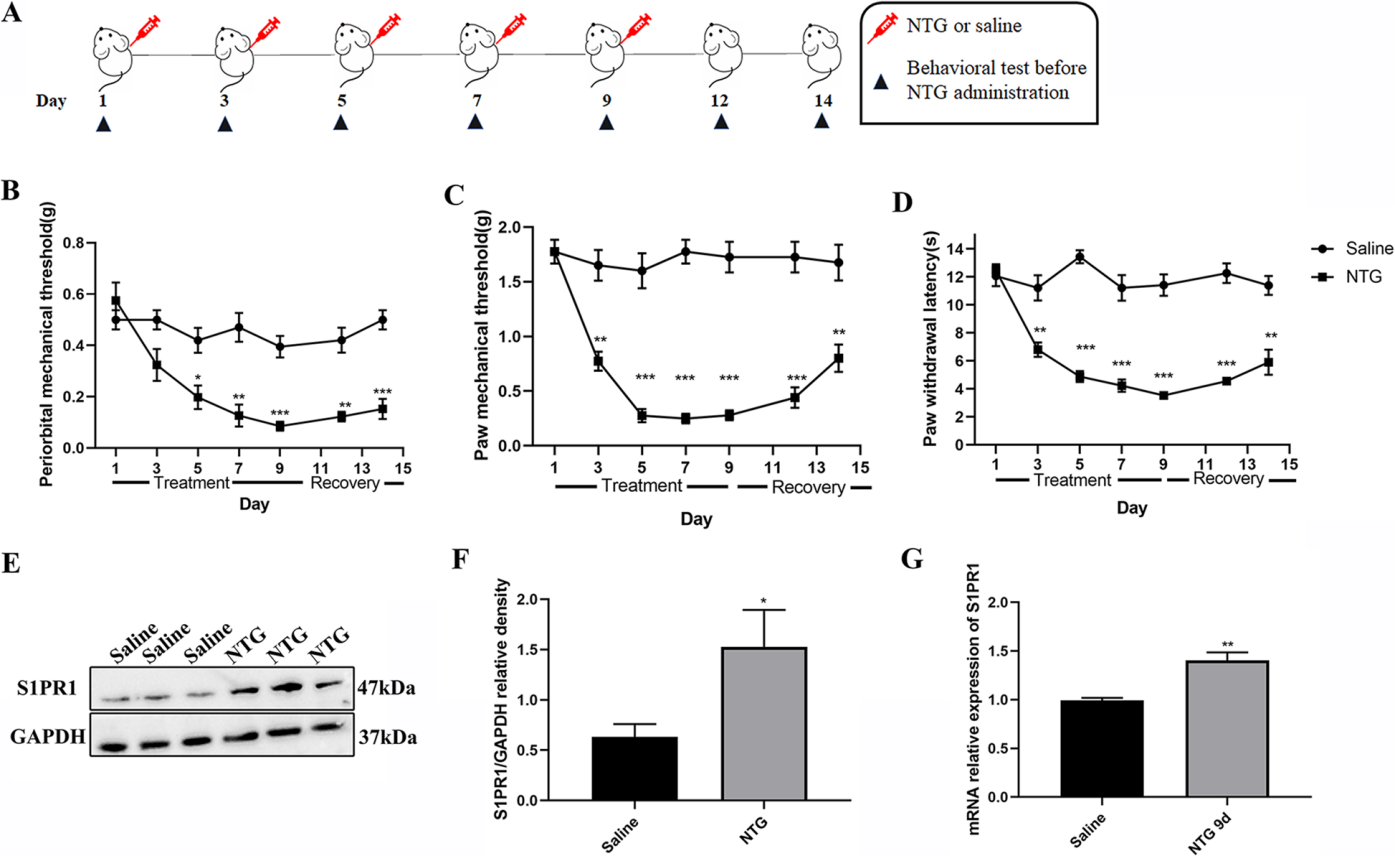
Upregulation of S1PR1 expression at mRNA and protein levels after chronic NTG injection. **A** Timeline of NTG or saline administration and behavioral testing. **B-D** Compared with the Saline group, the mechanical pain thresholds of periorbital in the NTG group gradually decreased from fifth day (*n* = 8/group, *p < 0.05, vs Saline); the mechanical and thermal pain thresholds of hindpaw in the NTG group gradually decreased from third day (*n* = 8/group, **p < 0.01, vs Saline); *p < 0.05, **p < 0.01 and ***p < 0.001 compared with the Saline group. **E, F** The expression of S1PR1 at protein level increased in the TCC following NTG injection for 9 days (*n* = 6/group, *p < 0.05, vs Saline). **G** The mRNA relative expression of S1PR1 increased in the NTG (9d) group compare with the Saline group (*n* = 6/group, **p < 0.01, vs Saline)

As previously described, to better mimic the clinical features of chronic migraine, mice were given NTG (10 mg/kg, *i.p.*) every other day for 5 times [27]. As shown in Fig. 1A-D, repeated NTG injection induced a gradual decrease in mechanical and thermal pain threshold in male mice, which was most obvious on the 9th day, and these decreases remained at 14th day after stopping the injection of NTG. To explore whether the expression of S1PR1 changed in the TCC area after repeated NTG administration, we compared the group of repeated NTG intermittent injections for 9 days with the saline group. We found that in the NTG group the expression of S1PR1 in mRNA and protein levels was significantly higher than the saline group after the fifth administration of NTG (Fig. 1E-G).

In order to further observe the distribution and cellular localization of S1PR1 in the TCC area, we co-stained S1PR1 with Iba1(Ionized calcium-binding adaptor molecule, a microglia marker), GFAP (Glial fibrillary acidic protein, an astrocyte marker), and NeuN (Neuronal nuclei, a neuronal marker) in the NTG group. Double immunofluorescence staining showed microglia, astrocytes and neurons in the TCC region could express S1PR1 [see Additional file 1].

### Blocking S1PR1 signaling attenuated mechanical and thermal hyperalgesia caused by chronic NTG administration

**Fig. 2:**
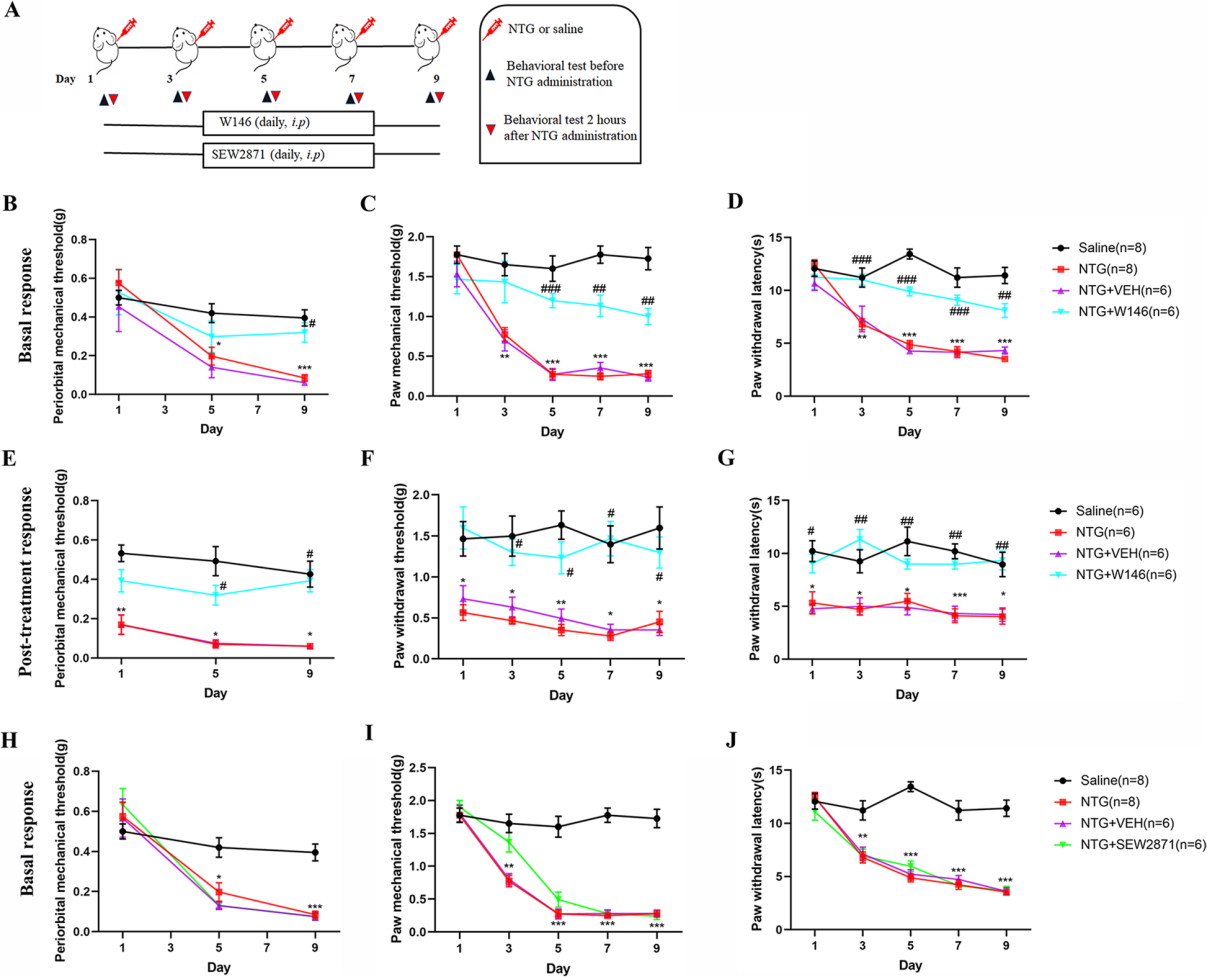
Blocking S1PR1 signaling attenuated mechanical or thermal hyperalgesia caused by chronic NTG administration. **A** Timeline of drug administration and behavioral testing. **B-C** Repeated daily injection of W146 before NTG could prevented basal mechanical and thermal hyperalgesia of periorbital **(B)** and hindpaw **(C, D)**. **E–G** post—treatment responses were assessed 2 h after NTG administration. W146 could attenuate NTG-triggered acute hyperalgesia. Two-way ANOVA and Bonferroni post hoc test analysis; *p < 0.05, **p < 0.01 and ***p < 0.001 compared with the Saline group; ^#^*p* < 0.05, ^##^*p* < 0.01 and ^###^*p* < 0.001 compared with the NTG group. **H-J** Repeated daily injection of SEW2871 (a S1PR1 agonist) before NTG did not prevent basal hyperalgesia of periorbital **(H)** and hindpaw **(I-J)**. Two-way ANOVA and Bonferroni post hoc test analysis; *p < 0.05, **p < 0.01 and ***p < 0.001 compared with the Saline group

To determine whether S1PR1 in TCC area was involved in the development and maintenance of hyperalgesia caused by chronic NTG administration, W146, a S1PR1 specific antagonist, was administered by intraperitoneal injection (Fig. 2A). In order to reduce the fear of filaments in mice, we measured periorbital nociception threshold on days 1, 5, and 9. The basal responses were evaluated before NTG, and the post-treatment responses were evaluated 2 h after NTG administration. We found that chronic treatment with W146 could significantly reduce basal mechanical or thermal hyperalgesia compared with NTG group (Fig. 2B-D). Furthermore, we also observed that W146 can also relieve acute hypersensitivity caused by NTG injection (Fig. 2E-G).

Instead, we observed that chronic administration of S1PR1 receptor agonist, SEW2871, could not attenuate thermal and mechanical allodynia caused by repeated NTG administration (Fig. 2H-J).

### Blocking S1PR1 signaling reduced the expression of CGRP and c-fos in the TCC

**Fig. 3:**
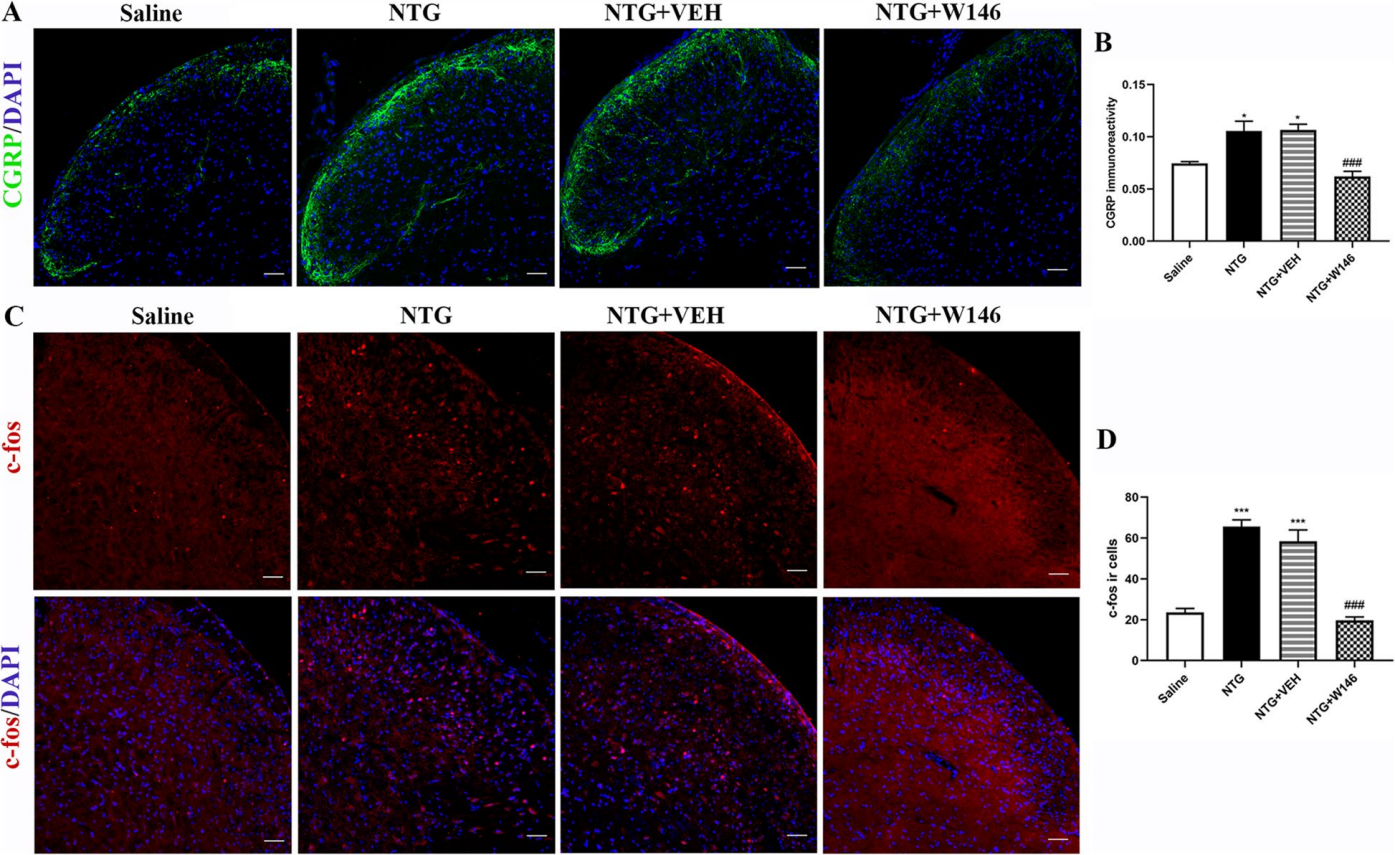
Blocking S1PR1 signaling reduced the expression of CGRP and c-fos in the TCC. **A, B** Representative immunofluorescence staining images **(A)** and quantitative analysis of CGRP in different groups **(B)**. Repeated daily injection of W146 reversed the increased immunoreactivity of CGRP induced by chronic NTG administration. *n* = 3/group; six sections from each mouse; *p < 0.05 vs. Saline group; ^###^*p* < 0.001 vs. NTG group. Scale bars = 50 μm. **C, D** Double immunofluorescence staining images of c-fos (red) and DAPI (blue) in the TCC area, these results indicated daily administration of W146 decreased c-fos ir cells. *n* = 3/group; ***p < 0.001 compared with the Saline group; ^###^*p* < 0.001 for the NTG + W146 group compared with the NTG group. Scale bars = 50 μm

As an important biomarker of migraine, CGRP plays an important role in the onset and maintenance of migraine [6, 36]. Compared with the saline group, repeated NTG administration induced a significant increase of the CGRP immunoreactivity in the superficial area of TCC (*p* < 0.05; Fig. 3A). However, chronic administration of W146 prevented the increase in CGRP immunostaining induced by NTG in the NTG + W146 group (*p* < 0.001, Fig. 3B).

c-fos is used as a valuable indicator of neuronal activation in nociceptive pathways, often marked by immunofluorescence staining in the study of CM [37]. Two hours after the last administration of NTG or vehicle, TCC tissues were taken to observe the immunoreactive cells of c-fos. The number of c-fos positive cells in the NTG group was significantly higher than that the saline group (*p* < 0.001). It also could be observed that the number of c-fos positive cells decreased in the NTG + W146 group (*p* < 0.001; Fig. 3C, D). Altogether, these results further confirmed that blocking S1PR1 signaling could alleviate chronic NTG-induced central sensitization.

### Blocking S1PR1 signaling suppressed NTG-induced microglia activation in the TCC area

**Fig. 4:**
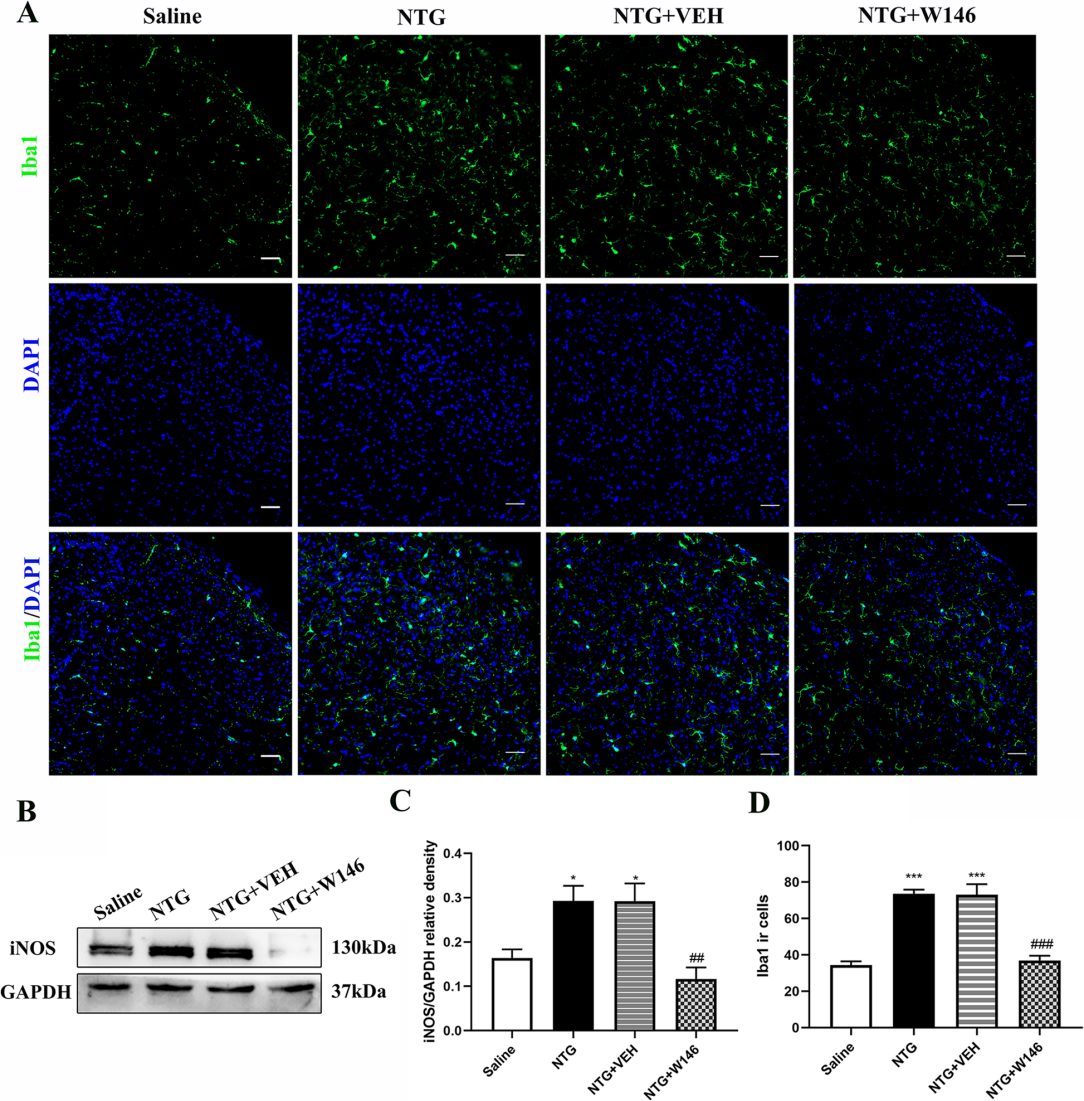
Blocking S1PR1 signaling suppressed NTG-induced microglia activation in the TCC area**. A** Representative immunofluorescence images of Iba1 in the TCC for the Saline, NTG, NTG + VEH and NTG + W146 group under an 10 × objective lens. **B-C** Western blots showed the changes in iNOS protein levels among the Saline, NTG, NTG + VEH and NTG + W146 group. These results indicated daily administration of W146 decreased the expression of iNOS. *n* = 6 per group; *p < 0.05 compared with the Saline group; ^###^*p* < 0.001 compared with the NTG group. **D** Quantification of Iba1-positive cells. These results indicated daily administration of W146 decreased Iba1 ir cells. *n* = 3 per group; ***p < 0.001 compared with the Saline group; ^###^*p* < 0.001 for the NTG + W146 group compared with the NTG group

It has been reported that chronic NTG stimulation induces an increase of microglia activity in the TCC [26]. In order to verify the hypothesis that chronic administration of W146 may reduce chronic NTG-induced central sensitization by inhibiting the activation of microglia. At first, we observed the changes of microglia by immunofluorescence labeling Iba1. Consistent with our previous reports, repeated NTG administration increased the number of Iba1 positive cells in the TCC area (*p* < 0.001). W146 markedly suppressed NTG induced upregulation of microglia in the TCC (*p* < 0.001, Fig. 4A, D).

iNOS (inducible nitric oxide synthase), a marker of M1 type microglia, was also evaluated in this study. The expression of iNOS in the chronic NTG model increased significantly (*p* < 0.05; Fig. 4B). However, the increase of iNOS were significantly suppressed in the NTG + W146 group (*p* < 0.01; Fig. 4B, C).

### Blocking S1PR1 attenuates LPS-induced increase of pSTAT3 in BV2 microglia in vitro

**Fig. 5:**
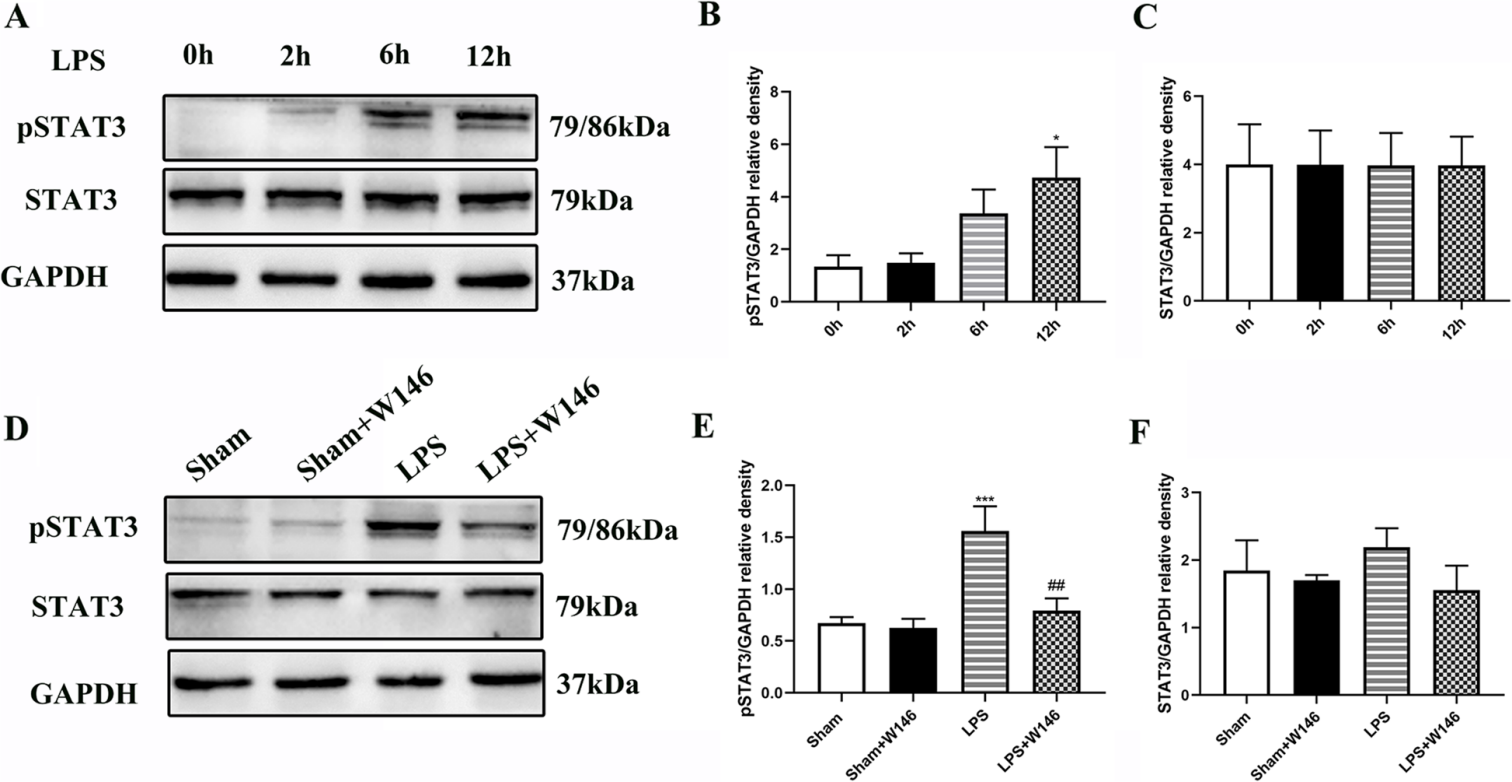
Blocking S1PR1 attenuates LPS-induced increase of pSTAT3 in BV2 microglia in vitro*.* **A-C** Representative western blots results showed that LPS induced an increase of pSTAT3 and this increase was time-dependent, most obvious after 12 h of LPS stimulation in BV2 cells **(A, B)**; However, LPS did not change the expression of STAT3 **(A, C);** Data were presented as mean ± SEM of three independent experiments; *p < 0.05 compared with LPS 0 h. **D-F** Pre-incubation of W146 before LPS stimulation could reduce the expression of pSTAT3, but had no effect on STAT3. Data were presented as mean ± SEM of three independent experiments; ***p < 0.001 vs. Sham group; ^#^*p* < 0.05, ^##^*p* < 0.01 vs. LPS group

STAT3, an important regulator of inflammatory gene expression, was involved in microglia-mediated inflammatory responses to various central nervous system stimulation [24, 25]. In order to explore whether the STAT3 signaling pathway is downstream of S1PR1, we first studied BV2 cells in vitro. We found that after 0.1 µg/ml LPS stimulation, phosphorylated STAT3 (pSTAT3) increased in a time-dependent manner, which was most obvious at 12 h of LPS stimulation (*p* < 0.05, Fig. 5A, B). The total amount of STAT3 did not change significantly (Fig. 5C).

However, with the pre-incubation of W146 before LPS stimulation, LPS had no effect on the level of pSTAT3 (*p* < 0.01, Fig. 5D-F). In addition, W146 treatment alone did not affect the expression of pSTAT3.

### Inhibition of STAT3 activity attenuated chronic NTG-induced mechanical and thermal hyperalgesia

**Fig. 6:**
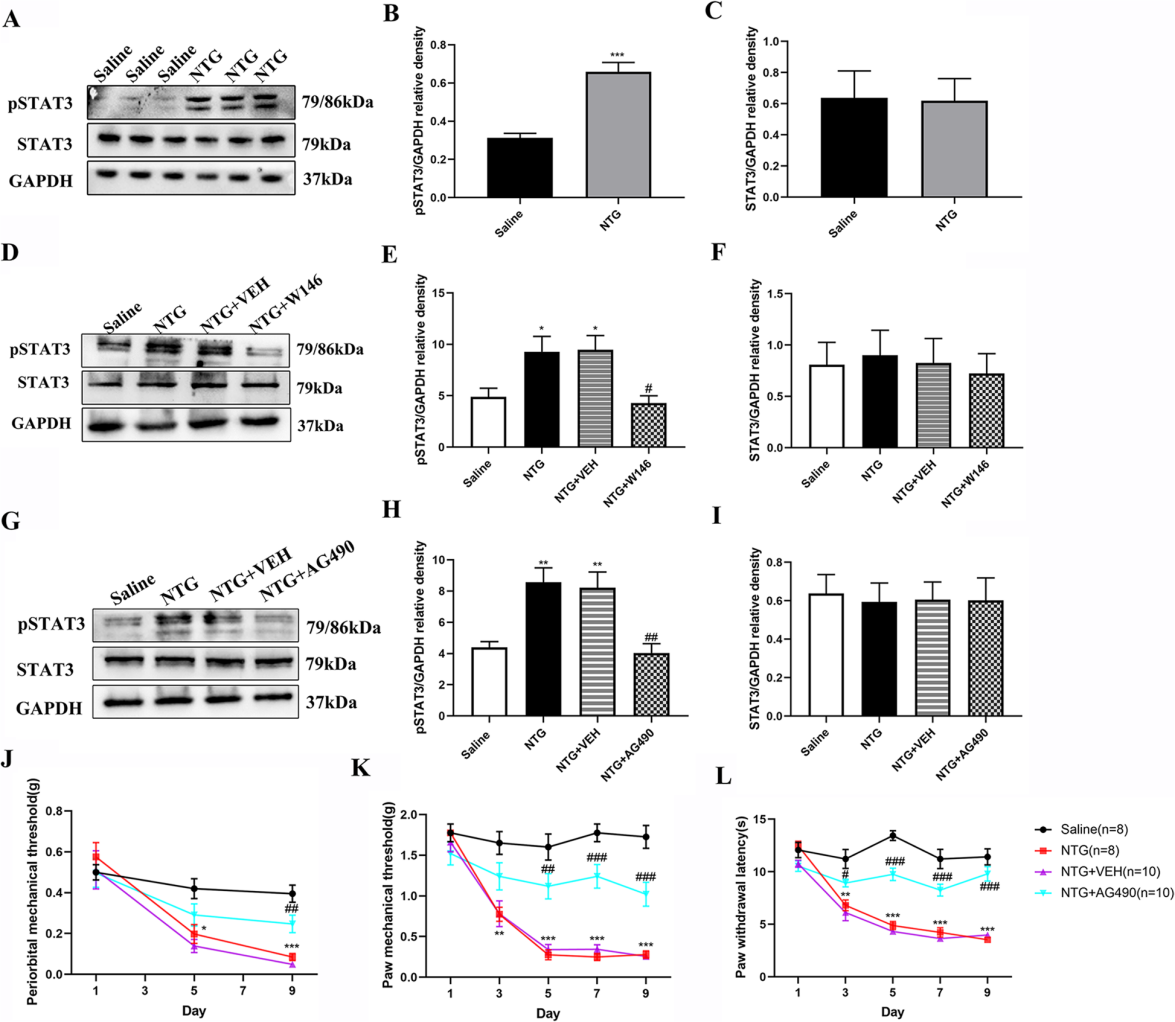
Inhibition of STAT3 activity attenuated chronic NTG-induced mechanical and thermal hyperalgesia. **A-C** Representative immunoblot showed that the expression level of pSTAT3 was up-regulated after repeated NTG administration **(A, B)**, while the expression of STAT3 remained unchanged **(A, C)**. The band intensities of pSTAT3/STAT3 were compared with GAPDH, respectively. *n* = 6 per group. ***p < 0.001 compared with the Saline group. **D, F** Representative immunoblot showed that the expression level of pSTAT3 decreased in the NTG + W146 group, but the expression level of STAT3 remained unchanged. *n* = 6 per group. * *p* < 0.05 compared with the Saline group. ^#^ *p* < 0.05 compared with the NTG group. **G-I** The STAT3 signaling inhibitor AG490 reversed the increase of pSTAT3(**G, H**) after repeated NTG injection but did not affect STAT3(**G, I**) in the TCC area. *n* = 6 per group. ** *p* < 0.01 compared with the Saline group. ^##^*p* < 0.01 compared with the NTG group. **J-L** Baseline thermal and mechanical pain threshold response of mice. Two-way ANOVA and Bonferroni post hoc test analysis; *p < 0.05, **p < 0.01 and ***p < 0.001 compared with the Saline group; ^#^*p* < 0.05, ^##^*p* < 0.01 and ^###^*p* < 0.001 for the NTG + AG490 group compared with the NTG group

To further elucidate whether STAT3 signaling pathway was involved in central sensitization induced by chronic NTG injection, we first detected the expression of pSTAT3 by western blot. We found that after repeated injections of NTG, the expression of pSTAT3 increased significantly compared with the saline group (*p* < 0.001, Fig. 6A, B), and the expression of STAT3 did not change markedly (Fig. 6A, C). Repeated administration of W146 for 9 days could reverse the increase of pSTAT3, (*p* < 0.05; Fig. 6D-F).

We further observed the effect of blocking the STAT3 signaling pathway on chronic NTG-induced pain behavior. As mentioned earlier, chronic intermittent NTG administration gradually induced mechanical and thermal hyperalgesia. In addition, we found that treatment with AG490 for 9 days increased the paw withdrawal latency and the mechanical threshold from the fifth day (Fig. 6J-L).

### Inhibition of STAT3 activity suppressed the expression level of CGRP and c-fos

**Fig. 7:**
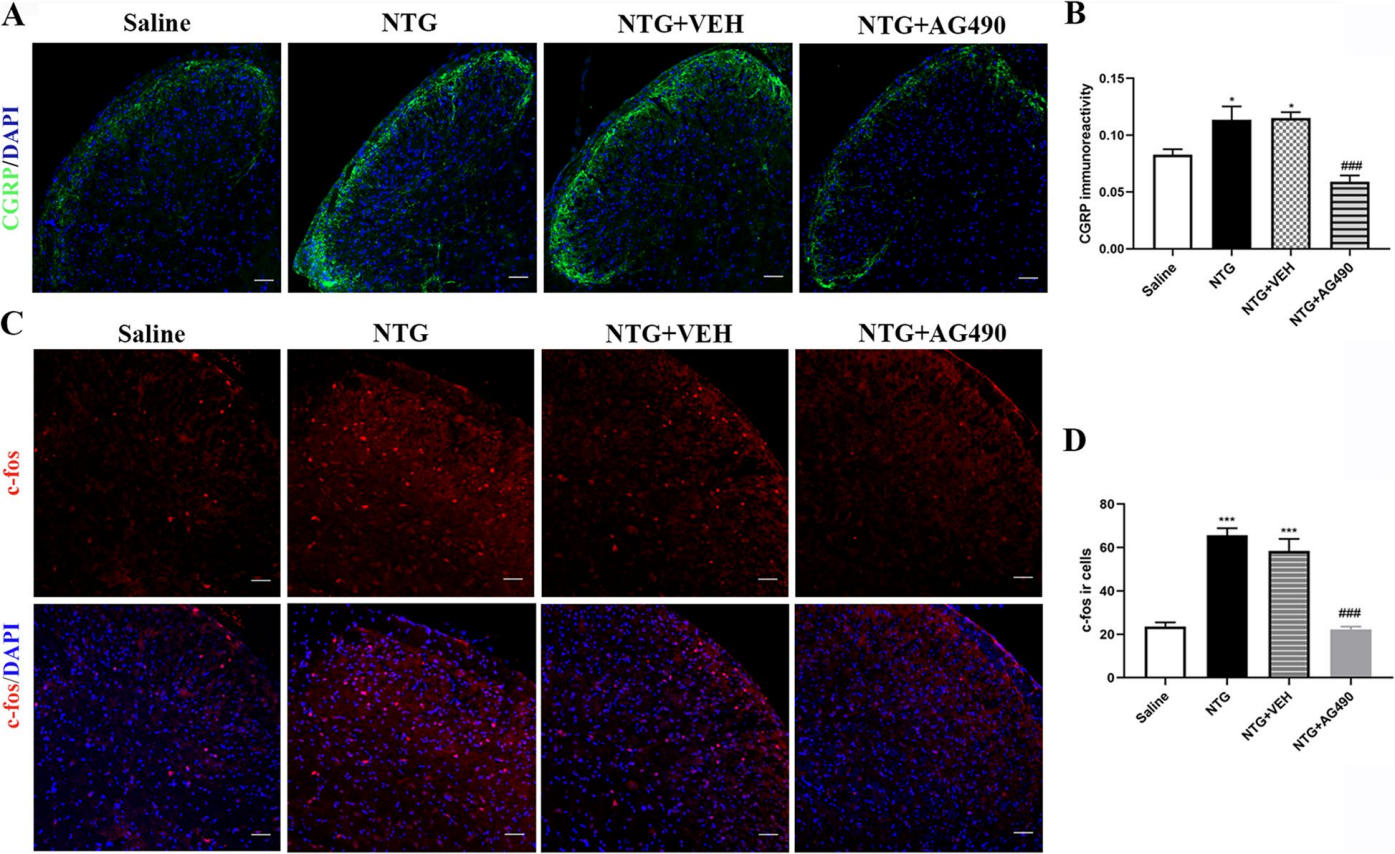
Inhibition of STAT3 activity could suppress the expression level of CGRP and c-fos. **A, B** Representative immunofluorescence staining images **(A)** and quantitative analysis of CGRP in the TCC area among the Saline, NTG, NTG + VEH and NTG + AG490 groups **(B)**. Repeated daily injection of AG490 inhibited the increased immunoreactivity of CGRP induced by chronic NTG administration. *n* = 4/group; *p < 0.05 vs. Saline group; ^###^*p* < 0.001 vs. NTG group. Scale bars = 50 μm. **C**, **D** Quantitative analysis of c-fos immunoreactive (ir) cells indicated daily administration of AG490 decreased c-fos ir cells. *n* = 4/group; ***p < 0.001 compared with the Saline group; ^###^*p* < 0.001 for the NTG + AG490 group compared with the NTG group. Scale bars = 50 μm

In addition, we quantified the expression of CGRP and c-fos by immunofluorescence staining. We found that AG490 could significantly reduce the expression of CGRP in the superficial area of TCC compared with the NTG and NTG + VEH group (*p* < 0.001, Fig. 7A, B). Consistent with the decrease in CGRP, chronic administration of AG490 significantly reduced the number of c-fos positive cells compared with the NTG and NTG + VEH group (*p* < 0.001, Fig. 7C, D). Altogether, these results revealed that blocking STAT3 signal could alleviate the central sensitization caused by chronic NTG administration.

## Discussion

There are several highlights in this study. (1) First, chronic NTG intermittent injection for 9 days induced increased expression of S1PR1 at the level of protein and mRNA in the TCC area. (2) Blocking the S1PR1 receptor could attenuate chronic NTG-induced hyperalgesia and the expression of CGRP and c-fos. (3) Blocking S1PR1 signaling suppressed NTG-induced microglia activation in the TCC area. (4) As a downstream signaling pathway of S1PR1, STAT3 pathway was involved in the central sensitization of CM. Blocking the STAT3 signaling pathway could alleviate central sensitization in a chronic NTG model.

S1PR1, a G protein-coupled receptor, is an important role of S1P in central sensitization of neuropathic pain, which distributed in peripheral and central pain modulatory system, such as spinal nerve roots and spinal dorsal horns [15, 16, 38]. Previous studies have found that S1PR1 expression in the dorsal horn of the spinal cord can be up-regulated when receiving noxious stimuli [16, 39]. TCC is an important secondary neuron for central sensitization of migraine. In this study, it was found for the first time that chronic intermittent NTG injection induced up-regulation of S1PR1 at the TCC area, and S1PR1 was co-labeled with microglia, astrocytes, and neurons in the NTG group, which suggests that S1PR1 is involved in central sensitization of CM.

S1PR1 has become a new target for the treatment of central nervous system diseases, including multiple sclerosis and Parkinson’s disease [40–42]. Although early studies reported that activating S1PR1 has analgesic effects, emerging evidence implicated genetic and pharmacological inhibition of S1PR1 could alleviate mechanical and thermal hyperalgesia in various neuropathic pain models [43, 44]. One study reported that W146, a specific inhibitor of S1PR1, can block S1P-induced hyperalgesia, but W140 (specific inhibitor of S1PR3) did not block the hyperalgesia [38]. A recent study reported that targeting S1PR1 with S1PR1 antagonists or RNA silencing could block morphine-induced hyperalgesia. Moreover, W146 can block the development of tolerance to the anti-allodynic effects of morphine and prevent prolonged morphine-induced neuropathic pain in mice with neuropathic pain [45]. In this study, we found that daily intraperitoneal administration of W146 could relieve acute and chronic hyperalgesia induced by NTG, but SEW2871 could not improve hyperalgesia. These results suggest that blocking S1PR1 may also become a new target for clinical treatment of chronic migraine.

Accumulated evidence indicates that microglia contribute to the development of central sensitization and chronic pain via neuron-glia cross-talk [8]. Minocycline, a microglia inhibitor, could reduce NTG-induced basic mechanical hyperalgesia and Iba1 immunoreactivity staining [26]. In addition, administration of microglial purinoceptor (P2X4R, P2X7R and P2Y12R) antagonists could also significantly improve the development of chronic migraine [10–12]. Studies in neuropathic pain have found that inhibition of S1PR1 can significantly reduce the activation of astrocytes and microglia in the spinal dorsal horn [30, 45]. However, the role of astrocytes in the chronic migraine model induced by nitroglycerin is unclear, and further research is needed. In addition, in vitro studies have confirmed that S1PR1 antagonist could promote the neuroprotective effect of microglia [42, 46]. In this study, we also found that repeated NTG injections could activate microglia at the TCC, which is characterized by an increase in the number of microglia and the expression of iNOS. Continuous administration of W146 could inhibit the activation of microglia at the TCC site. From these data, we conclude that inhibiting the S1PR1 activity of microglia may be a key target to alleviate NTG-induced central sensitization.

STAT3 is an important type of transcription factor and mainly activated by the direct phosphorylation of tyrosine (705) and forms a dimer, followed by nuclear translocation, DNA binding, and regulation of a variety of cytokines [24]. A previous study reported that blocking of STAT3 signaling pathway could attenuate ipsilateral mechanical and thermal hypersensitivity and the mirror-image mechanical allodynia induced by peripheral nerve injury [24]. Although the S1P and STAT3 signaling pathways are mostly reported in cancer research, a recent preclinical study of multiple sclerosis suggests that S1PR1 in microglia promotes the activation of downstream STAT3 signaling pathways in microglia [40, 47, 48]. In this study, the expression of pSTAT3 at the TCC site was up-regulated after repeated NTG administration. Previous studies have shown that AG490, as a JAK2 inhibitor, can significantly inhibit the STAT3 signaling pathway [49, 50]. In this study we found that the thermal and mechanical pain threshold increased and the expression of CGRP and c-fos decreased after continuous administration of AG490. our results provide the first evidence that the STAT3 pathway participates in the central sensitization of CM. In addition, we further found that the STAT3 pathway is an important downstream pathway of S1PR1 through in vivo and in vitro experiments.

Our previous studies indicate that microglia are involved in the central sensitization of chronic migraine, thus this study focused on effects of W146 mainly mediated by microglial signaling in chronic NTG model. The effects of S1PR1 on neurons or astrocyte need further investigation. In addition, Previous studies implicated that S1PR1 on glia cells modulated various downstream signaling pathway in neuropathic pain model, including MAPK pathway or NLRP3 inflammasome [51, 52]. But this research focuses on the STAT3 signaling pathway, alternate pathways were not evaluated in this study.

## Conclusions

In conclusion, our study demonstrated that blocking S1PR1 activity with W146 could alleviate chronic NTG-induced hypersensitivity and reduce the expression of CGRP and c-fos in the TCC area. We also found that W146 inhibited NTG-induced microglial activity in the TCC. The anti-hyperalgesic effect of W146 is associated with the STAT3 pathway in chronic NTG model. These findings suggest that S1PR1 in the TCC may be a new target for the treatment of CM.

## Supplementary Information

**Additional file 1: Figure S1** Location of S1PR1 in TCC.

## Data Availability

All experimental data for this study are available from the corresponding author on reasonable request.

## Abbreviations

CM: Chronic migraine
NTG: Nitroglycerin
S1P: Sphingosine-1-phophate
S1PR1: Sphingosine-1 Phosphate Receptor 1
TCC: Trigeminocervical complex
STAT3: Signal transducers and activators of transcription 3
LPS: Lipopolysaccharide
pSTAT3: Phosphorylated signal transducers and activators of transcription 3
qRT-PCR: Quantitative real-time polymerase chain reaction
SDS-PAGE: Sodium dodecyl sulfate polyacrylamide
DOMS: Dimethyl sulfoxide
CGRP: Calcitonin gene related peptide
SEM: Standard error of the mean
GAPDH: Glyceraldehyde-3-phosphate dehydrogenase
Iba1: Ionized calcium binding adaptor molecule 1
iNOS: Inducible nitric oxide synthase
GFAP: Glial fibrillary acidic protein
NeuN: Neuronal nuclei

## References

[1] Ashina M, Katsarava Z, Do TP. et al. 2021. Migraine: epidemiology and systems of care. Lancet. London, England. 10.1016/S0140-6736(20)32160-7 10.1016/S0140-6736(20)32160-7 33773613

[2] Manack AN, Buse DC, Lipton RB (2011). Chronic migraine: epidemiology and disease burden. Curr Pain Headache R..

[3] Headache Classification Committee of the International Headache Society (IHS) (2018). The International Classification of Headache Disorders, 3rd edition. Cephalalgia.

[4] Torres-Ferrús M, Ursitti F, Alpuente A (2020). From transformation to chronification of migraine: pathophysiological and clinical aspects. The journal of headache and pain.

[5] Woolf CJ (2011). Central sensitization: implications for the diagnosis and treatment of pain. Pain.

[6] Su M, Yu S (2018). Chronic migraine: A process of dysmodulation and sensitization. Mol Pain.

[7] Aurora SK (2009). Spectrum of illness: understanding biological patterns and relationships in chronic migraine. Neurology.

[8] Chen G, Zhang YQ, Qadri YJ (2018). Microglia in Pain: Detrimental and Protective Roles in Pathogenesis and Resolution of Pain. Neuron.

[9] Zhou X, Liang J, Wang J (2020). Up-regulation of astrocyte excitatory amino acid transporter 2 alleviates central sensitization in a rat model of chronic migraine. J Neurochem.

[10] Jiang L, Zhang Y, Jing F (2021). P2X7R-mediated autophagic impairment contributes to central sensitization in a chronic migraine model with recurrent nitroglycerin stimulation in mice. J Neuroinflamm.

[11] Jing F, Zhang Y, Long T (2019). P2Y12 receptor mediates microglial activation via RhoA/ROCK pathway in the trigeminal nucleus caudalis in a mouse model of chronic migraine. J Neuroinflamm.

[12] Long T, He W, Pan Q (2020). Microglia P2X4R-BDNF signalling contributes to central sensitization in a recurrent nitroglycerin-induced chronic migraine model. The journal of headache and pain.

[13] Spiegel S, Milstien S (2003). Sphingosine-1-phosphate: an enigmatic signalling lipid. Nature reviews. Molecular cell biology.

[14] Coste O, Brenneis C, Linke B (2008). Sphingosine 1-phosphate modulates spinal nociceptive processing. The Journal of biological chemistry.

[15] Sim-Selley LJ, Goforth PB, Mba MU (2009). Sphingosine-1-phosphate receptors mediate neuromodulatory functions in the CNS. J Neurochem.

[16] Chen Z, Doyle TM, Luongo L (2019). Sphingosine-1-phosphate receptor 1 activation in astrocytes contributes to neuropathic pain. P Natl Acad Sci Usa.

[17] Stockstill K, Doyle TM, Yan X (2018). Dysregulation of sphingolipid metabolism contributes to bortezomib-induced neuropathic pain. The Journal of experimental medicine.

[18] Karunakaran I, Alam S, Jayagopi S (2019). Neural sphingosine 1-phosphate accumulation activates microglia and links impaired autophagy and inflammation. Glia.

[19] Noda H, Takeuchi H, Mizuno T (2013). Fingolimod phosphate promotes the neuroprotective effects of microglia. J Neuroimmunol.

[20] Cipriani R, Chara JC, Rodríguez-Antigüedad A (2015). FTY720 attenuates excitotoxicity and neuroinflammation. J Neuroinflamm.

[21] Guo Y, Gan X, Zhou H (2020). Fingolimod suppressed the chronic unpredictable mild stress-induced depressive-like behaviors via affecting microglial and NLRP3 inflammasome activation. Life Sci.

[22] Pyne NJ, Pyne S (2017) Sphingosine 1-Phosphate Receptor 1 Signaling in Mammalian Cells. Molecules (Basel, Switzerland) 22(3):344. 10.3390/molecules22030344 10.3390/molecules22030344 PMC6155263 28241498

[23] Yu H, Pardoll D, Jove R (2009). STATs in cancer inflammation and immunity: a leading role for STAT3. Nature reviews. Cancer.

[24] Dominguez E, Rivat C, Pommier B (2008). JAK/STAT3 pathway is activated in spinal cord microglia after peripheral nerve injury and contributes to neuropathic pain development in rat. J Neurochem.

[25] Hu Z, Deng N, Liu K (2020). CNTF-STAT3-IL-6 Axis Mediates Neuroinflammatory Cascade across Schwann Cell-Neuron-Microglia. Cell Rep.

[26] Long T, He W, Pan Q (2018). Microglia P2X4 receptor contributes to central sensitization following recurrent nitroglycerin stimulation. J Neuroinflamm.

[27] Pradhan AA, Smith ML, Mcguire B (2014). Characterization of a novel model of chronic migraine. Pain.

[28] Wang Y, Pan Q, Tian R (2021). Repeated oxytocin prevents central sensitization by regulating synaptic plasticity via oxytocin receptor in a chronic migraine mouse model. J Headache Pain.

[29] Pettingill P, Weir GA, Wei T (2019). A causal role for TRESK loss of function in migraine mechanisms. Brain: a journal of neurology.

[30] Doolen S, Iannitti T, Donahue RR (2018). Fingolimod reduces neuropathic pain behaviors in a mouse model of multiple sclerosis by a sphingosine-1 phosphate receptor 1-dependent inhibition of central sensitization in the dorsal horn. Pain.

[31] Gao F, Gao Y, Meng F (2018). The Sphingosine 1-Phosphate Analogue FTY720 Alleviates Seizure-induced Overexpression of P-Glycoprotein in Rat Hippocampus. Basic Clin Pharmacol.

[32] Romero-Reyes M, Ye Y (2013). Pearls and pitfalls in experimental in vivo models of headache: conscious behavioral research. Cephalalgia: an international journal of headache.

[33] Vuralli D, Wattiez AS, Russo AF (2019). Behavioral and cognitive animal models in headache research. The journal of headache and pain.

[34] He W, Long T, Pan Q (2019). Microglial NLRP3 inflammasome activation mediates IL-1β release and contributes to central sensitization in a recurrent nitroglycerin-induced migraine model. J Neuroinflamm.

[35] Zheng Z, Zeng YZ, Ren K (2019). S1P promotes inflammation-induced tube formation by HLECs via the S1PR1/NF-κB pathway. Int Immunopharmacol.

[36] Riesco N, Cernuda-Morollón E, Pascual J (2017). Neuropeptides as a Marker for Chronic Headache. Curr Pain Headache R.

[37] Harriott AM, Strother LC, Vila-Pueyo M (2019). Animal models of migraine and experimental techniques used to examine trigeminal sensory processing. The journal of headache and pain.

[38] Xie W, Strong JA, Kays J (2012). Knockdown of the sphingosine-1-phosphate receptor S1PR1 reduces pain behaviors induced by local inflammation of the rat sensory ganglion. Neurosci Lett.

[39] Cuzzocrea S, Doyle T, Campolo M (2018). Sphingosine 1-Phosphate Receptor Subtype 1 as a Therapeutic Target for Brain Trauma. J Neurotraum.

[40] Tsai HC, Nguyen K, Hashemi E (2019). Myeloid sphingosine-1-phosphate receptor 1 is important for CNS autoimmunity and neuroinflammation. J Autoimmun.

[41] Zhao P, Yang X, Yang L (2017). Neuroprotective effects of fingolimod in mouse models of Parkinson’s disease. FASEB journal: official publication of the Federation of American Societies for Experimental Biology.

[42] Zhong L, Jiang X, Zhu Z (2019). Lipid transporter Spns2 promotes microglia pro-inflammatory activation in response to amyloid-beta peptide. Glia.

[43] Grenald SA, Doyle TM, Zhang H (2017). Targeting the S1P/S1PR1 axis mitigates cancer-induced bone pain and neuroinflammation. Pain.

[44] Lee BJ, Kim JY, Cho HJ (2020). Sphingosine 1-phosphate receptor modulation attenuate mechanical allodynia in mouse model of chronic complex regional pain syndrome by suppressing pathogenic astrocyte activation. Region Anesth Pain M.

[45] Doyle TM, Janes K, Chen Z (2020). Activation of sphingosine-1-phosphate receptor subtype 1 in the central nervous system contributes to morphine-induced hyperalgesia and antinociceptive tolerance in rodents. Pain.

[46] Zahiri D, Burow P, Großmann C (1868). (2021) Sphingosine-1-phosphate induces migration of microglial cells via activation of volume-sensitive anion channels, ATP secretion and activation of purinergic receptors. Biochimica et biophysica acta. Molecular cell research.

[47] Estrada-Bernal A, Palanichamy K, Ray Chaudhury A (2012). Induction of brain tumor stem cell apoptosis by FTY720: a potential therapeutic agent for glioblastoma. Neuro-Oncology.

[48] Liu Y, Deng J, Wang L (2012). S1PR1 is an effective target to block STAT3 signaling in activated B cell-like diffuse large B-cell lymphoma. Blood.

[49] Li CD, Zhao JY, Chen JL (2019). Mechanism of the JAK2/STAT3-CAV-1-NR2B signaling pathway in painful diabetic neuropathy. Endocrine.

[50] Tsuda M, Kohro Y, Yano T (2011). JAK-STAT3 pathway regulates spinal astrocyte proliferation and neuropathic pain maintenance in rats. Brain: a journal of neurology.

[51] Janes K, Little JW, Li C (2014). The development and maintenance of paclitaxel-induced neuropathic pain require activation of the sphingosine 1-phosphate receptor subtype 1. The Journal of biological chemistry.

[52] Doyle TM, Chen Z, Durante M (2019). Activation of Sphingosine-1-Phosphate Receptor 1 in the Spinal Cord Produces Mechanohypersensitivity Through the Activation of Inflammasome and IL-1β Pathway. The journal of pain.

