# Supplementary material for: Sphingosine-1 phosphate receptor 1 contributes to central sensitization in recurrent nitroglycerin-induced chronic migraine model
Source: J Headache Pain. 2022 Feb 10;23(1):25. doi: 10.1186/s10194-022-01397-w (PMC8903593; doi:10.1186/s10194-022-01397-w)
Supplement: Supplementary file 1 — Additional file 1: Figure S1 Location of S1PR1 in TCC. [file 10194_2022_1397_MOESM1_ESM.docx]

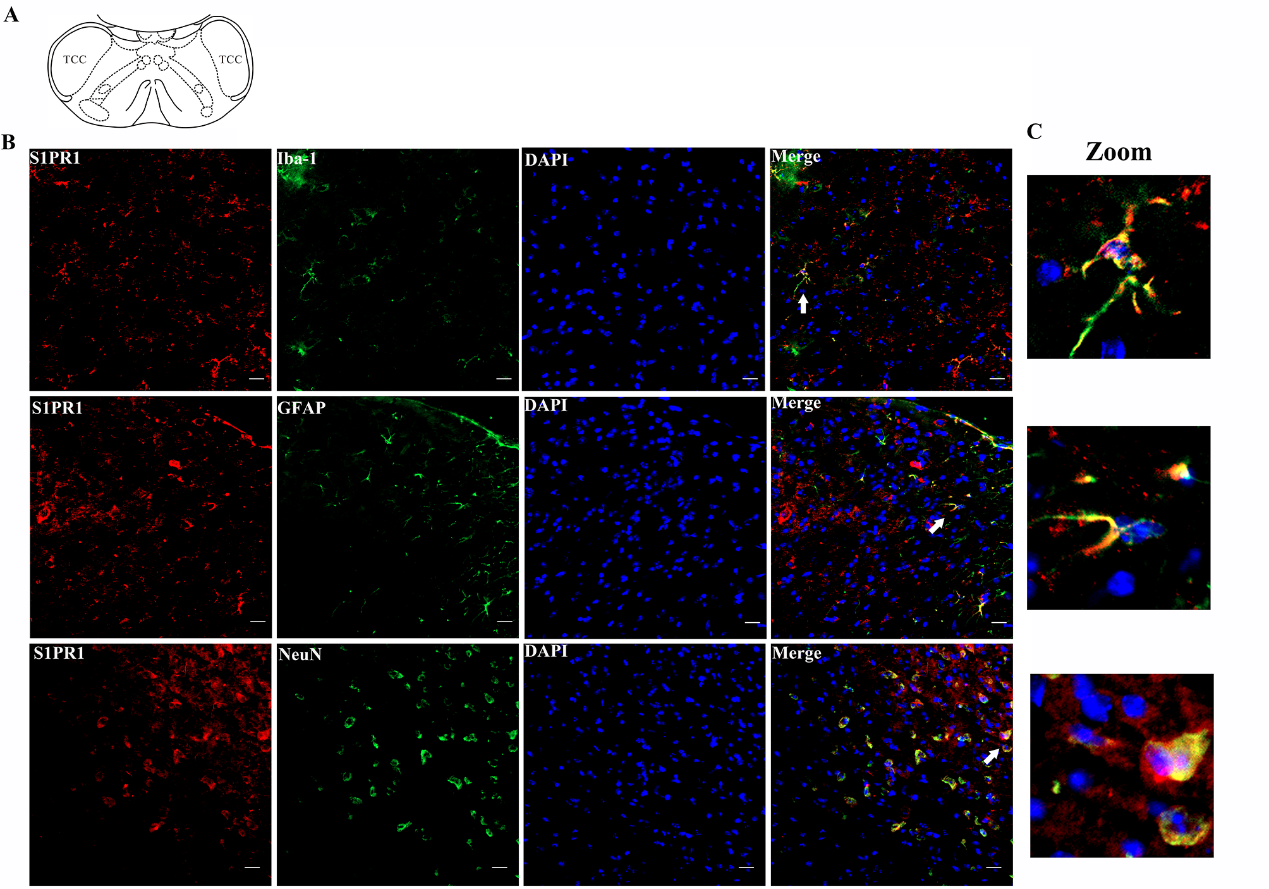


**Additional Fig. 1** Localization of S1PR1 in the TCC. **a** Representative image of the coronal plane of the mouse TCC area. **b** Representative images of immunofluorescence staining showing the co-localization of S1PR1(red) with microglia (Iba1), astrocytes (GFAP) and neurons (NeuN) in the mice with NTG administration. Scale bar = 20 μm **c** Enlarged co-staining picture.
